# Effects of COVID-19 on Japanese medical students’ knowledge and attitudes toward e-learning in relation to performance on achievement tests

**DOI:** 10.1371/journal.pone.0265356

**Published:** 2022-03-14

**Authors:** Miwa Sekine, Makino Watanabe, Shuko Nojiri, Tsutomu Suzuki, Yuji Nishizaki, Yuichi Tomiki, Takao Okada

**Affiliations:** 1 Division of Medical Education, Juntendo University Faculty of Medicine, Tokyo, Japan; 2 Medical Technology Innovation Center, Juntendo University Faculty of Medicine, Tokyo, Japan; 3 Department of Physiology, Juntendo University Faculty of Medicine, Tokyo, Japan; 4 Department of Respiratory Medicine, Juntendo University Faculty of Medicine, Tokyo, Japan; 5 Department of Coloproctological Surgery, Juntendo University Faculty of Medicine, Tokyo, Japan; Satyawati College (Eve.), University of Delhi, INDIA

## Abstract

The COVID-19 pandemic forced many educational institutions to turn to electronic learning to allow education to continue under the stay-at-home orders/requests that were commonly instituted in early 2020. In this cross-sectional study, we evaluated the effects of the COVID-19 pandemic on medical education in terms of students’ attitudes toward online classes and their online accessibility; additionally, we examined the impacts of any disruption caused by the pandemic on achievement test performance based on the test results. The participants were 674 students (412 in pre-clinical, 262 in clinical) at Juntendo University Faculty of Medicine; descriptive analysis was used to examine the respondents’ characteristics and responses. The majority of respondents (54.2%) preferred asynchronous classes. Mann–Whitney U tests revealed that while pre-clinical students preferred asynchronous classes significantly more than clinical students (39.6%, *p* < .001), students who preferred face-to-face classes had significantly higher total achievement test scores (*U* = 1082, *p* = .021, *r* = .22). To examine the impacts of pandemic-induced changes in learning, we conducted Kruskal–Wallis tests and found that the 2020 and 2021 scores were significantly higher than those over the last three years. These results suggest that while medical students may have experienced challenges adapting to electronic learning, the impact of this means of study on their performance on achievement tests was relatively low. Our study found that if possible, face-to-face classes are preferable in an electronic learning environment. However, the benefit of asynchronous classes, such as those that allow multiple viewings, should continue to be recognized even after the pandemic.

## Introduction

The pandemic arising from the COVID-19 disease, which emerged in November 2019 [1] in Wuhan, China, forced many educational institutions to halt their activities, and stay-at-home orders/requests were issued in many cities worldwide. As of May 1, 2021, more than 151 million cases had been documented all over the world, causing approximately 3.1 million deaths [2–5], with 592,709 positive cases and more than 10,293 deaths in Japan [6]. This highly contagious virus required social distancing to be imposed, and the operations of medical schools were affected by these rules. However, the same circumstances inspired creative innovation and ingenuity in the responses developed to this unprecedented crisis [7, 8].

Since the onset of the pandemic, numerous educational institutions have rapidly adapted to online or other forms of instruction to continue education [9], taking account of the risk of infection to students, staff, faculty, and teachers [10, 11]. Not only have institutions improvised entire curricula [12], constructed novel class forms [7, 13, 14], often they were required to select and install online education systems [15, 16]. Additionally, educators and administrative personnel needed to install appropriate systems and learn how to use these systems, often via faculty development [17]. It cannot be denied that there are both advantages and disadvantages to doing online classes using conventional systems, such as YouTube, learning management systems [18, 19], Zoom (Zoom Video Communications, Inc., San Jose, CA, USA), and other electronic means. However, the attitudes of medical students toward e-learning as well as accessibility may vary greatly by geographic region [20–22].

Clinical clerkships in Japanese medical schools incorporate a residency for fourth-year medical students, wherein they join a medical team that performs actual medical procedures and conducts clinical care. Before this step, students undergo a performance assessment, the Objective Structured Clinical Examination, and a knowledge assessment administered through computer-based testing using item response theory [23] are conducted at the end of pre-clerkship courses (in the fourth year), which includes basic clinical knowledge, introduction to clinical medicine, clinical skills, and clinical reasoning. These tests, developed by the Common Achievement Tests Organization [http://www.cato.umin.jp/], were implemented in 2005 as standardized tests [24, 25].

Japan has 82 medical schools, 31 of which are private. Juntendo University is one of these private medical schools. We prepared portable Wi-Fi for students who needed it, as well as a hybrid online/face-to-face system that allowed a small number of students to come to school and attend classes in a location with better internet connectivity, which those who could commute to the university could take advantage of. Many educational institutions turned to online lectures during the pandemic, and students attended them from home. Medical students, even those undergoing clinical training, were required to take online courses to avoid contact with high-risk patients (including pre-clinical students). Numerous studies have explored students’ mental health during the pandemic as well as the impacts of pandemic-induced changes on the medical education system. However, the attitude of medical students toward e-learning and academic performance, especially related to their performance in computer-based achievement tests, remain unknown. This study aimed to understand the impact of the COVID-19 pandemic on medical students and to identify any negative effects on their education using questionnaire surveys and the results of achievement tests. In particular, the study attempted to determine whether the pandemic had any negative impact on the academic performance based on the results of the computer-based achievement test. The findings of this study will help to provide a better understanding of effective educational responses and will be beneficial to educators and educational institutions across the world that may face similar challenges even after the end of the COVID-19 pandemic.

## Methods

### Study design

A cross-sectional study was conducted at Juntendo University Faculty of Medicine, Tokyo, Japan, using a self-administered questionnaire survey of second- to sixth-year medical students that was distributed from June to July 2020. The completed questionnaires were collected by one of the authors to ensure confidentiality and prevent bias. Official examination scores of computer-based testing from 2017 to 2021 were collected with the written consent of students and the approval of the university ethical review board (No. 2020187). All study procedures were conducted according to the principles of World Medical Association Declaration of Helsinki.

### Participants

Paper-based questionnaires were provided to all 686 students (both male and female) in their second to sixth year at Juntendo University Faculty of Medicine from June to July 2020. Respondents who did not provide written informed consent documentation and freshman, who had no previous experience with Juntendo University classes, were excluded. Subsequently, 674 completed, valid questionnaires were collected (a response rate of 98%), 132 of which, those from fourth-year students, were used to conduct a comparison analysis with their test scores. Second- to fourth-year students were categorized as pre-clinical students; fifth- and sixth-year students were categorized as clinical students because they have passed the requisite examinations to become student doctors in their fourth year. Because the fourth-year students had not yet taken the requisite examinations when this study was conducted, they were considered pre-clinical students. The participants were mostly male. A summary of the distribution of responses is given in Table 1.

**Table 1:** Basic characteristics of participants (n = 674).

Education Level	Total (%)	Gender		Age
		Male (%)	Female (%)	Mean ± S.D.
**Pre-Clinical**				
**Second year**	141(20.9)	77(11.4)	64(9.5)	19.96 ± 1.28
**Third year**	139(20.6)	96(14.2)	43(6.4)	20.88 ± 0.92
**Fourth year**	132(19.6)	88(13.1)	44(6.5)	21.85 ± 0.9
**Clinical**				
**Fifth year**	132(19.6)	91(13.5)	41(6.1)	23.04 ± 1.03
**Sixth year**	130(19.3)	93(13.8)	37(5.5)	24.05 ± 1.16
**Total**	674(100)	445(66)	229(34)	21.91 ± 1.81

Age calculated as of June 30, 2020.

This questionnaire was designed to survey and assess internet access and attitudes toward online classes. Students were informed of this study’s purpose, guaranteed anonymity, and informed that their answers would have no effect on their grades; further, they were instructed that they could opt out of the project anytime if they no longer wished to participate.

### Testing

The computer-based achievement (CBT) test for the fourth-year students consisted of 320 test questions (multiple-choice and extended matching items) selected randomly from a pool of validated test questions used by medical schools in Japan. Students were evaluated on a total of 240 out of those 320, with 80 being new trial questions under evaluation for future use. The questions were designed to evaluate basic clinical knowledge.

### Statistical analysis

The collected data were managed using Microsoft Excel. IBM SPSS Statistics for Windows, Version 27.0. (Armonk, NY: IBM Corp) was used for statistical analysis. Descriptive analyses were used to examine respondents’ characteristics and responses. Frequencies and percentages were used for categorical variables, and means and standard deviations were used for continuous variables. The Pearson chi-square test was used to determine the correlation of variables based on gender or clinical/pre-clinical student groups. The correlation was further investigated using z test for the population proportion. The p-value threshold for significance was adjusted for multiple comparisons using the Bonferroni correction. Fourth-year students’ questionnaires were used to conduct comparison analysis with their test scores in 2020. The Shapiro–Wilk test and box plots were used to assess the normality of test scores. Using the test results for normality, the Mann–Whitney U tests was used to determine the significance of the differences in test scores based on questionnaire answers, and Kruskal–Wallis tests were used to examine the mean rank differences in CBT scores by year. The scores were further explored through the use of Dunn–Bonferroni tests. *r* and *ε^2^* were used to determine the effect sizes for the Mann–Whitney U tests and the Kruskal–Wallis tests, respectively. The threshold for statistical significance was set at *p* < .05 for each analysis.

## Results

### Technology usage and availability

A large portion of students (84%) had connections to the internet at their residences even before the pandemic. A small fraction (6.4%) of students had unsatisfactory internet access. There were no significant differences in device usage between male and female; however, tablet usage was higher among clinical students than pre-clinical students (*p* < .001).

### Medical students’ attitude toward online classes

The majority of respondents preferred asynchronous classes (54.2%). Female students rated the advantage of “being able to review” materials (*p =* .008) and “being able to watch multiple times” (*p =* .013) significantly more often than male students. A large proportion of medical students preferred asynchronous classes. Female students considered the “ease of keeping daily routine” (20.3%), “being able to meet friends” (27%), and “ease of asking questions” (7.1%) to be the main advantages of face-to-face classes. Male students found face-to-face classes’ “strict attendance requirement” to be a disadvantage (28.6%).

Clinical students reported that face-to-face classes had the advantage that they “give sense of presence/involvement” (*p =* .016), while pre-clinical students found face-to-face classes caused “difficulty staying focused” (*p* < .001).

Asynchronous classes were preferred by many students, especially pre-clinical students (*X*^2^ (4, N = 674) = 91.293, *p* < .001) with the reason being “convenience in scheduling class,” “being able to rewind and review,” “being able to watch multiple times,” and “being able to plan study schedule,” with the main disadvantage being “difficulty in keeping daily routine.” Synchronous classes such as Zoom were even less popular than face-to-face classes, and pre-clinical students reported that they found it difficult that synchronous classes made them “bound by class schedule” while clinical students found synchronous classes to be preferable, as they found that they created an “ease of asking questions” that was less recognized among pre-clinical students.

The characteristics of and differences between male and female participants are summarized in Table 2A, and the differences between pre-clinical students and clinical students are presented in Table 2B.

**Table 2:** Internet accessibility, device usage, and attitude toward online classes during the COVID-19 pandemic.

		(A) Gender			(B) *Level of Education*		
*Variables (Multiple response)*	Total (%)	Male (%)	Female (%)	*p*	Pre-Clinical (%)	Clinical (%)	*p*
	n = 674	n = 445	n = 229		n = 412	n = 262	
**Which device do you use to take classes?**							
Apple Macintosh	195(28.9)	130(19.3)	65(9.6)	.766	130(19.3)	65(9.6)	.055
Windows	269(39.9)	171(25.4)	98(14.5)	.314	169(25.1)	100(14.8)	.432
Tablet	260(38.6)	168(24.9)	92(13.6)	.601	137(20.3)	123(18.2) ***	< .001
Smart phone	78(11.6)	51(7.6)	27(4)	.932	49(7.3)	29(4.3)	.729
**What is your internet availability?**							
Internet network available at home	566(84)	368(54.6)	198(29.4)	.710	341(50.6)	225(33.4)	.418
Use mobile carrier data	33(4.9)	21(3.1)	12(1.8)	.838	17(2.5)	16(2.4)	.259
Set up internet system specifically for classes	32(4.7)	17(2.5)	15(2.2)	.138	24(3.6)	8(1.2)	.093
Borrowed Wi-Fi router from university	7(1)	5(0.7)	2(0.3)	.732	5(0.7)	2(0.3)	.564
Using Wi-Fi elsewhere (e.g., public access)	20(3)	11(1.6)	9(1.3)	.326	14(2.1)	6(0.9)	.395
Dissatisfactory	43(6.4)	31(4.6)	12(1.8)	.332	31(4.6)	12(1.8)	.118
**How would you prefer that classes be delivered?**							
Face-to-face	134(19.9)	78(11.6)	56(8.3) *	.031	74(11)	60(8.9)	.122
Asynchronous (e.g., On-demand)	365(54.2)	242(35.9)	123(18.2)	.913	267(39.6) ***	98(14.5)	< .001
Synchronous (e.g., Zoom)	39(5.8)	22(3.3)	17(2.5)	.187	14(2.1)	25(3.7) **	.001
No preference	138(20.5)	103(15.3) *	35(5.2)	.018	57(8.5)	81(12) ***	< .001
**What are the advantages of face-to-face classes?**							
Ease of keeping daily routine	346(51.3)	209(31)	137(20.3) **	.002	217(32.2)	129(19.1)	.395
Being able to meet friends	484(71.8)	302(44.8)	182(27) **	.002	307(45.5)	177(26.3)	.051
Give sense of presence/involvement	216(32)	133(19.7)	83(12.3)	.110	118(17.5)	98(14.5) *	.016
Ease of asking questions	113(16.8)	65(9.6)	48(7.1) *	.041	62(9.2)	51(7.6)	.131
Other	31(4.6)	23(3.4)	8(1.2)	.314	21(3.1)	10(1.5)	.442
**What are disadvantages of face-to-face classes?**							
Strict attendance requirement	258(38.3)	193(28.6) ***	65(9.6)	< .001	169(25.1)	89(13.2)	.076
Difficulty in staying focused	177(26.3)	111(16.5)	66(9.8)	.198	131(19.4) ***	46(6.8)	< .001
Difficulty in commuting to class	428(63.5)	277(41.1)	151(22.4)	.160	263(39)	165(24.5)	.918
Difficulty in asking questions Other	68(10.1)	47(7)	21(3.1)	.644	35(5.2)	33(4.9)	.079
	96(14.2)	59(8.8)	37(5.5)	.246	68(10.1) *	28(4.2)	.038
**What are the advantages of asynchronous classes?**							
Convenience in scheduling classes	580(86.1)	378(56.1)	202(30)	.081	375(55.6) ***	205(30.4)	< .001
Being able to rewind and review	384(57)	239(35.5)	145(21.5) **	.008	265(39.3) ***	119(17.7)	< .001
Being able to watch multiple times	433(64.2)	273(40.5)	160(23.7) *	.013	299(44.4) ***	134(19.9)	< .001
Being able to plan my study schedule	370(54.9)	237(35.2)	133(19.7)	.156	245(36.4) **	125(18.5)	.009
Other	53(7.9)	39(5.8)	14(2.1)	.246	33(4.9)	20(3)	.944
**What are the disadvantages of asynchronous classes?**							
Difficulty in keeping daily routine	289(42.9)	185(27.4)	104(15.4)	.265	196(29.1) **	93(13.8)	.002
No sense of presence/involvement	175(26)	116(17.2)	59(8.8)	.997	99(14.7)	76(11.3)	.137
Difficulty in staying focused	271(40.2)	169(25.1)	102(15.1)	.070	156(23.1)	115(17.1)	.101
Difficulty in asking questions	135(20)	81(12)	54(8)	.081	82(12.2)	53(7.9)	.894
Other	95(14.1)	75(11.1) **	20(3)	.005	64(9.5)	31(4.6)	.183
**What are the advantages of synchronous classes (e.g., via Zoom)?**							
Easy to have daily routine	288(42.7)	182(27)	106(15.7)	.191	179(26.6)	109(16.2)	.165
Being able to take classes with friends	111(16.5)	70(10.4)	41(6.1)	.495	56(8.3)	55(8.2) *	.037
Ease of staying focused	209(31)	129(19.1)	80(11.9)	.121	114(16.9)	95(14.1)	.088
Ease of asking questions	75(11.1)	54(8)	21(3.1)	.230	22(3.3)	53(7.9) ***	< .001
Other	65(9.6)	49(7.3)	16(2.4)	.086	50(7.4) **	15(2.2)	.002
**What are the disadvantages of synchronous classes (e.g., via Zoom)?**							
Difficulty in keeping daily routine	102(15.1)	76(11.3) *	26(3.9)	.043	61(9.1)	41(6.1)	.811
Bound by class schedule	421(62.5)	269(39.9)	152(22.6)	.166	278(41.2) ***	143(21.2)	< .001
Not being able to meet friends	194(28.8)	120(17.8)	74(11)	.167	127(18.8)	67(9.9)	.117
Difficulty in staying focused	101(15)	66(9.8)	35(5.2)	.918	54(8)	47(7)	.097
Difficulty in asking questions	101(15)	63(9.3)	38(5.6)	.431	70(10.4)	31(4.6)	.058
Other	63(9.3)	38(5.6)	25(3.7)	.335	41(6.1)	22(3.3)	.471

Results are based on two-sided tests. For each significant pair, asterisk appears in the category with the larger column proportion. Tests are adjusted for all pairwise comparisons per row using the Bonferroni correction. Percentages are parenthesis represent portion of valid total N. (S1 File. Questionnaire Raw Data.Sav)

Significance level for

* < .05

** < .01, and

*** < .001.

### Score correlation with technology availability and attitude toward online classes

We investigated the correlations between students’ attitudes toward online classes, the availability of technology, and the corresponding total score (correct answer rate) for computer-based achievement test (CBT) (Table 3). It was found that the scores had a significant correlation with the questionnaire answers. Students who preferred face-to-face classes had significantly higher scores overall (*p =* .021) relative to students who preferred other methods. Students who found “ease of keeping daily routine” total score (*p* = .046) to be advantages in face-to-face classes had significantly higher scores.

**Table 3:** Results of comparison of CBT score between students who selected answers listed below and students who selected otherwise.

	Answer	N	Mean	SD	Mean Rank	*U*	*Z*	*p*	*r*
*How would you prefer classes be delivered*?									
**Face-to-face**									
Total Score	Not Selected	108	79.56	8.64	64.52	1082.00	−2.315	.021	.22
	Selected	28	83.32	7.2	83.86				
*What are the advantages of face-to-face classes*?									
**Ease of keeping daily routine**									
Total Score	Not Selected	75	79.38	8.24	62.43	1832.00	−1.994	.046	.23
	Selected	61	81.51	8.69	75.97				

Each of the analyses are shown for the CBT score differences for those who selected particular choices and those who did not select it (total CBT score) as well as the results of Mann–Whitney U tests comparing the parameter estimates between the two groups. The mean differences are significant at the 0.05 level. (S2 File. CBT Raw Data.Sav).

Questions are in Italicized Text, Answers are in Bold Text, and CBT Score Subcategories are in Roman Text.

### Comparison of test scores between 2020 and 2021 and the previous three years (2017–2019)

**Fig 1:**
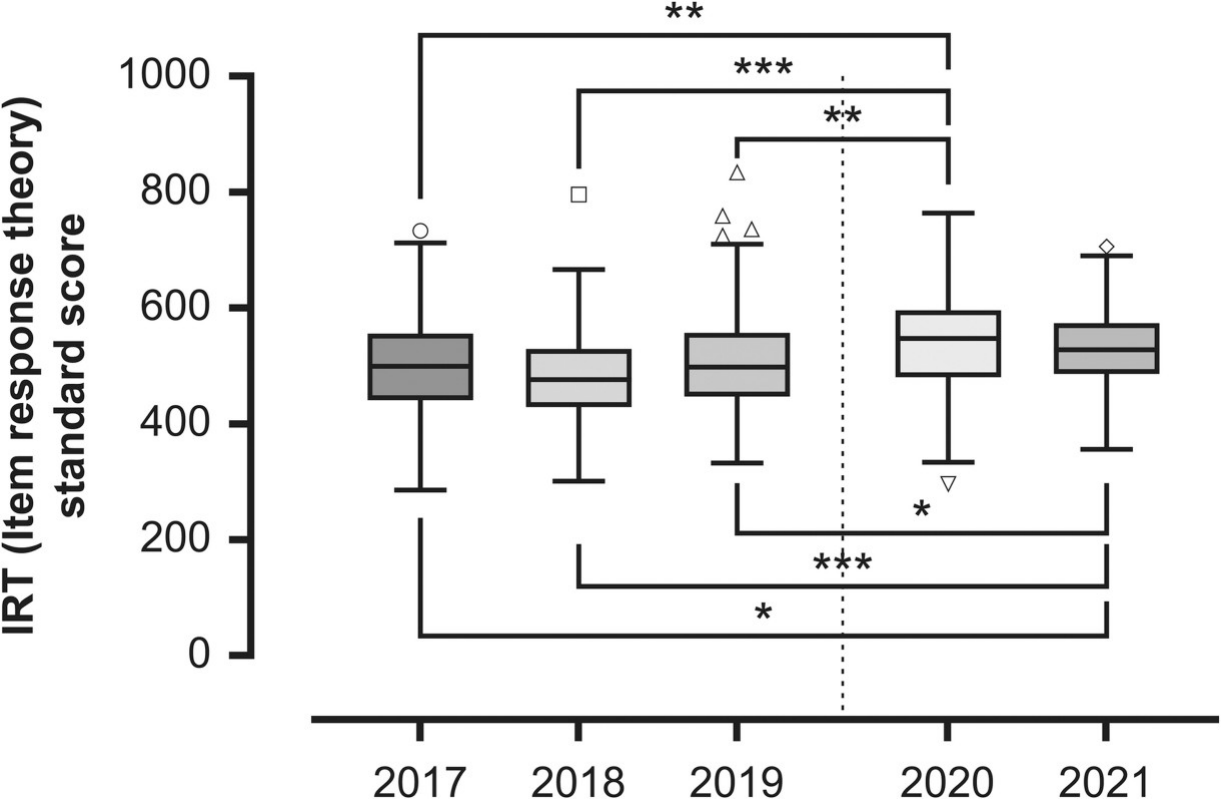
A comparison of IRT standard test scores between 2020 and 2021 and 2017 through 2019. Upper whisker represents maximum observation, lower whisker represents minimum observation, circle symbols represent outliers, upper line of box represents third quartile, middle line of box represents median, lower line of box represents first quartile, and x represents mean. Significance level for * < .05, ** < .01, and *** < .001.

**Fig 2:**
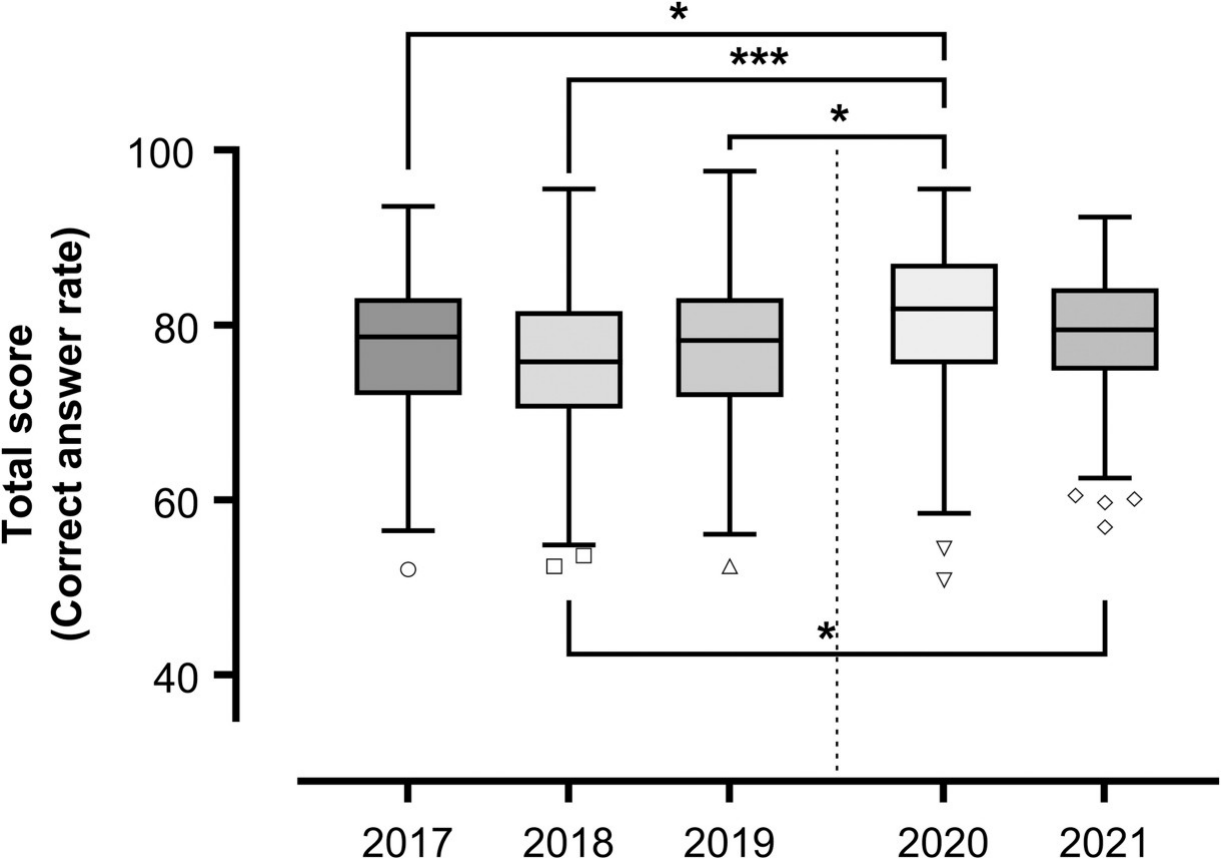
Total scores between 2020 and 2021 and 2017 through 2019. Upper whisker represents maximum observation, lower whisker represents minimum observation, circle symbols represent outliers, upper line of box represents third quartile, middle line of box represents median, lower line of box represents first quartile, and x represents mean. Significance level for * < .05, ** < .01, and *** < .001.

We used the Kruskal–Wallis test to analyze item response theory (IRT) standard scores and total scores (correct answer rate) in relation to other years wherein the examination format was the same as 2020 and 2021 to evaluate whether the pandemic had any effect on basic knowledge or test-taking ability. There were significant differences at the *p* < .05 level for the IRT standard score, *H* (4) = 42.74, *p* < .001, *ε*^*2*^ = .064; total score, *H* (4) = 27.87, *p* < .001, *ε*^*2*^ = .042, between the years. Post-hoc analyses using a Dunn–Bonferroni test indicated that test scores from 2020 and 2021 were significantly higher compared to the previous three years in the IRT standard scores: 2017 (*p* = .005), 2018 (*p* < .001), and 2019 (*p* = .004) against 2020; 2017 (*p* = .033), 2018 (*p* < .001), and 2019 (*p* = .027) against 2021; total score, 2017 (*p* = .016), 2018 (*p* < .001), 2019 (*p* = .014) against 2020; 2018 (*p* < .011) against 2021 (Figs 1 and 2). The results of the Kruskal–Wallis tests and a post-hoc analysis of the test scores are shown in Tables 4 and 5.

**Table 4:** Summary of Kruskal–Wallis test score comparison, 2017–2021.

Computer-based achievement (CBT) test	*χ^2^*	*df*	*p*	*ε^2^*
IRT Standard score	42.74	4	< .001	.064
Total Score	27.87	4	< .001	.042

Note. Mean difference is significant at the 0.05 level. (S2 File. CBT Raw Data.Sav).

**Table 5:** Dunn–Bonferroni test comparison of CBT sections between 2020 and 2021.

Computer-based achievement (CBT) test				*P*			
	N		Mean Rank	vs. 2020		vs. 2021	
IRT standard score	2017	133	312.82	.005	**	.033	*
	2018	126	262.32	< .001	***	< .001	***
	2019	132	311.25	.004	**	.027	*
	2020	136	394.54			1.000	
	2021	140	381.28	1.000			
Total score	2017	133	320.99	.016	*	1.000	
	2018	126	275.64	< .001	***	.011	*
	2019	132	320.01	.014	*	1.000	
	2020	136	395.14			.671	
	2021	140	352.68	.671			

Significance values have been adjusted by the Bonferroni correction for multiple tests. (S2 File. CBT Raw Data.Sav)

Significance level for

* < .05

** < .01, and

*** < .001.

## Discussion

This study explored medical students’ attitudes toward online classes and evaluated the effects of the pandemic on fourth-year students’ performance based on the CBT achievement test.

It was found that a large portion of students use computers rather than tablets or smart phones to take classes. Internet access was relatively high, but some students were not satisfied. Additionally, a significant difference was found in tablet use between pre-clinical students (33.4% of all pre-clinical students) and clinical students (47.1% of all clinical students), perhaps because students more commonly use tablets in clinical clerkship duties. Interestingly, when asked about their preference for class delivery, clinical students reported preferring asynchronous lectures less often than pre-clinical students, while pre-clinical students were more likely to prefer asynchronous lectures, such as on-demand classes. In asynchronous learning, students receive a lecture form through e-mail or a learning management system at times that can be convenient for students; additionally, the costs are relatively low, and the learning methods and schedules are flexible. On the other hand, this method may isolate students, and for many, a flexible schedule is less necessary [26]. New challenges have also emerged on the teaching side: some students do not turn on their cameras during synchronous classes [27], and teachers report an increase in the difficulty of evaluating students online [28], especially in competency-based medical education [29].

Synchronous online lectures, such as those conducted over Zoom, are bound by lecture times and their delivery is affected by students’ financial situation as well, such as in the internet speed, computer specifications, and in some cases, packet communication fees. This may be a reason why students do not turn on their cameras during classes [27], and it may also be a reason why many students prefer asynchronous classes, as well as not being satisfied with the online environment.

During the pandemic, many educational institutions have struggled to maintain their teaching quality. Both synchronous and asynchronous online classes may be a burden on both students and faculty, both financially and technically, and several sociological, financial, and technological barriers also affect students’ motivation [30–32]. Prior learning experiences have a positive effect on students’ evaluation and satisfaction with current online education [33]. If utilized properly, e-leaning can foster a range of abilities [34], such as creativity in children [35].

The correlation of CBT score with technology availability and attitude toward online classes shows that students who preferred face-to-face classes scored significantly higher than other students. This supports a report that indicated that students felt they were able to learn better in physical classrooms [22]. Further, students who reported that face-to-face classes made it easy to maintain their daily routines scored significantly higher on total score than students who answered otherwise. This shows that, if possible, face-to face classes that allow students to maintain their daily routines would be preferable to maximize education.

Although COVID-19 disrupted many lectures and training sessions, a comparison of the CBT results suggests that students were able to maintain their motivation at a relatively high level. Further exploration of Kruskal–Wallis test in each CBT sections (S1 Table) showed that five out of six sections were significantly higher in 2020 or 2021 (S2 Table). From the end of July to early August every year, Japanese medical schools hold the All-Japan Medical Students Athletic Meet, in which the majority of medical students participate. However, in 2020 and 2021, this tournament was canceled due to the pandemic, which led us to speculate that it is possible that scores were higher than in previous years because students were able to study more in the absence of preparation for the tournament. A comparison between the test results for participants and non-participants of this athletic meet over the past three years indicates that non-participants scored higher in 2019 (S3 Fig). This suggests that we cannot deny the possibility that students simply had more time to study due to the pandemic. There have been reports on the effects on mental health of the stay-home situation as well as isolation effects due to the spread of COVID-19 [36–38], there have also been reports that medical students’ motivations, attitudes, other mental health indicators, and even academic performance have remained relatively high [11, 15]. Further, unexpected positive effects of the pandemic, including innovative educational methods and increased motivation to learn, have also been reported [39, 40].

Since the pandemic began, numerous studies have been conducted on the difficulties that students and educators have encountered with e-learning as a result of the sudden shift to online learning. This study provides contributions to educators and to educational institutions to better understand students’ academic performance during COVID-19 as well as to provide better preparation for possible future educational crises similar to this pandemic. Consequently, we believe that further study of the long-term effects of the pandemic on students’ performance will be beneficial to the educational field.

## Conclusions

During the early phase of the pandemic, students and faculty alike experienced confusion and dread regarding online classes. Their dissatisfaction with the internet learning environment may have been due to sociological or financial problems during the pandemic, with the possibility of severe, disruptive financial change. A small number of dissatisfied students is never a small amount of casualties for an educational institution but can rather entail grave damage that signifies the need for prompt remedies. This study provides a glimpse of a way in which medical students are more resilient than was thought. This study concludes that while the pandemic caused disruptive effects on medical education, it is also the catalyst for unexpected innovation and inspiration, as well as the motivation to adapt and improve. Be that as it may, now that the situation has subsided and e-learning is the new standard style of education, we propose that educational facilities consider making recorded classes available for students to review multiple times, as well as providing additional assistance in cases of inadequate environments, such as by creating spaces for students to attend/watch classes if the internet environment is insufficient for learning.

In this study, we observed that most medical students had access to medical education via e-learning and were able to use electronic devices to attend classes in Juntendo University. However, our study was conducted in a single private medical school in a particular setting in a single country. Each country has exhibited its own characteristic response to the spread of COVID-19 in terms of allowance to the flow and movement of people and of course vaccination accessibility; therefore, individual countries’ regulations and technological infrastructure, economic situations, and so on are undeniably different. Thus, the results presented here might not be generalizable to other settings or countries and require further study. As of this writing (September 2021), the COVID-19 pandemic is ongoing, and for that reason, the study setting and its design were somewhat limited. As we face long-term restrictions of movement, coupled with social distancing, either voluntary or otherwise, we must take maximal measures to minimize the negative effects on education. We believe that our findings shed light on students’ educational performance and attitudes toward learning during the pandemic and could bear fruit used in further studies to investigate optimal pre-clinical and clinical education and facilitate students’ growth.

## Supporting information

S1 Fig Internet accessibility, device usage, and attitude toward online classes during the COVID-19 pandemic. (a). Comparisons Based on Gender. Percentage is of valid N (Male, Female).
(TIF)

S2 Fig Internet accessibility, device usage, and attitude toward online classes during the COVID-19 pandemic. (b). Comparisons Based on Students’ Education Level. Percentage is of valid N (clinical, pre-clinical).
(TIF)

S3 Fig Comparison of CBT score for the last 5 years between All-Japan students athletic meet participants and non-participants. Significance level for *< .05, **< .01. Error bars represent standard deviation.
(TIF)

S1 Table Test sections. (DOCX)

S2 Table Summary of the Kruskal–Wallis test of CBT sections test score comparison, 2017–2021. (DOCX)

S3 Table Dunn–Bonferroni test comparisons of CBT sections between 2020 and 2021. (DOCX)

S1 File Questionnaire raw data. (SAV)

S2 File CBT raw data. (SAV)
