# Supplementary figures and images for: Effects of COVID-19 on Japanese medical students’ knowledge and attitudes toward e-learning in relation to performance on achievement tests
Source: PLoS One. 2022 Mar 14;17(3):e0265356. doi: 10.1371/journal.pone.0265356 (PMC8920276; doi:10.1371/journal.pone.0265356)

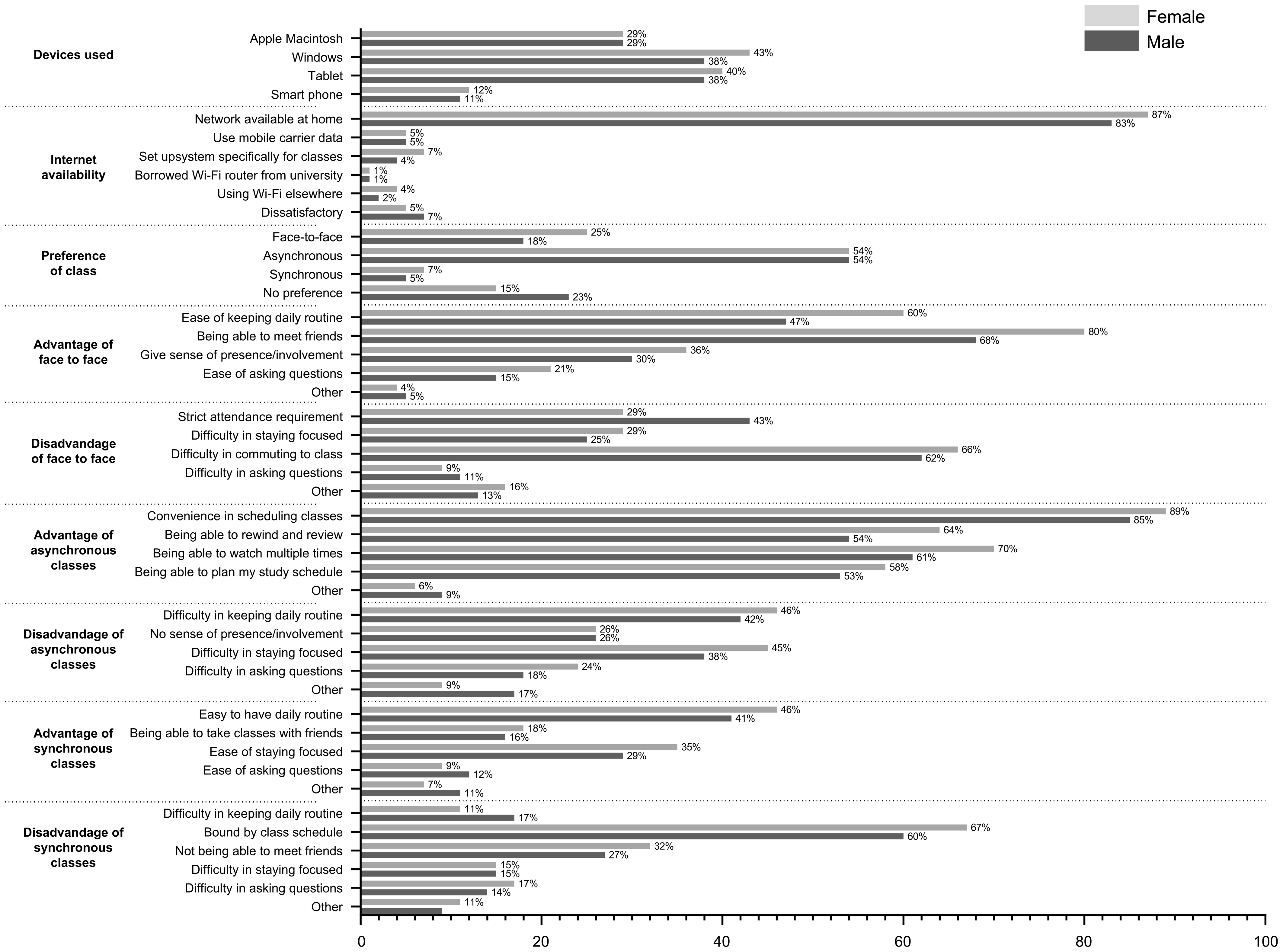

Supplement: S1 Fig — (a). Comparisons Based on Gender. Percentage is of valid N (Male, Female). (TIF) [file pone.0265356.s001.tif]

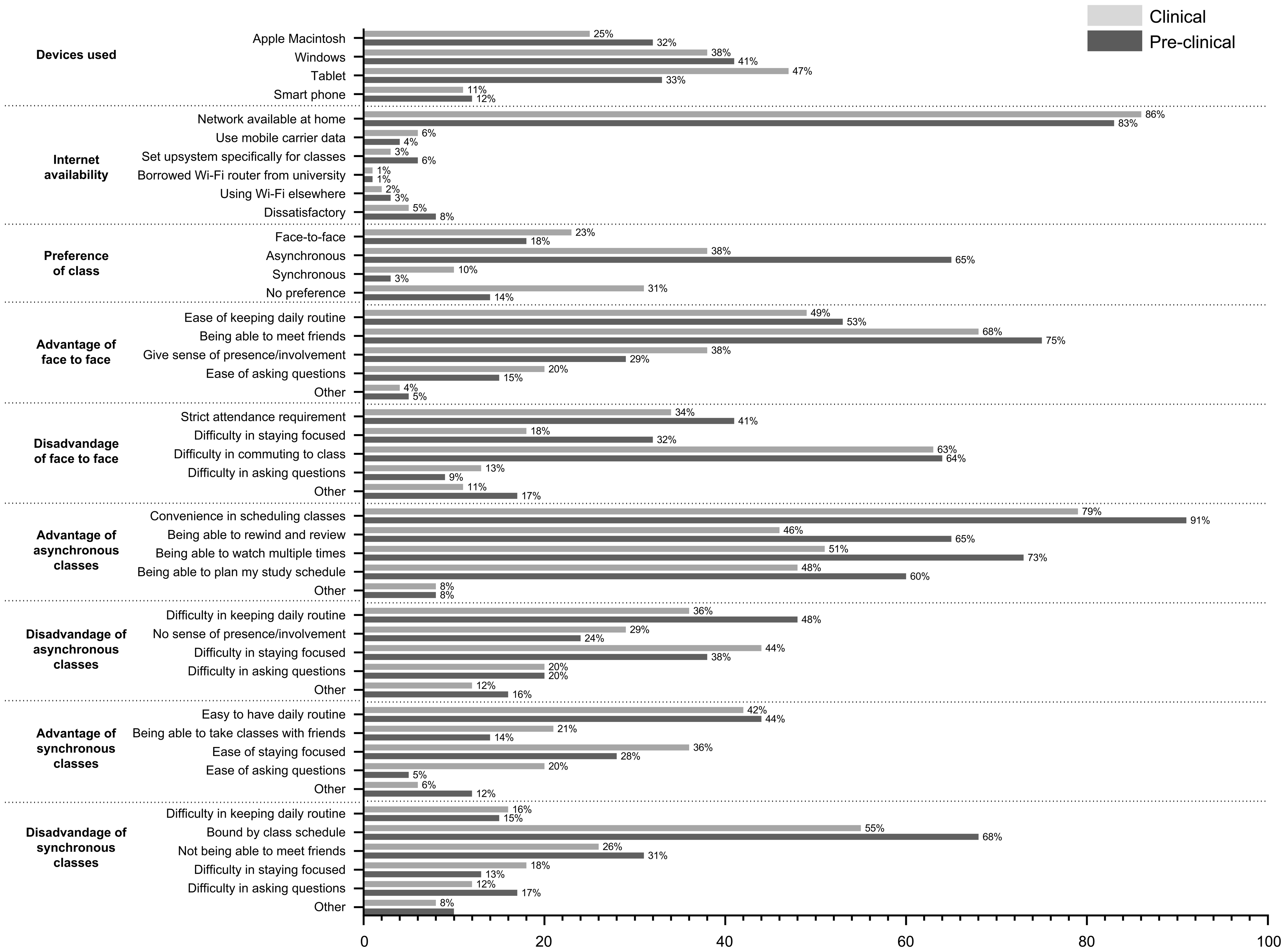

Supplement: S2 Fig — (b). Comparisons Based on Students’ Education Level. Percentage is of valid N (clinical, pre-clinical). (TIF) [file pone.0265356.s002.tif]

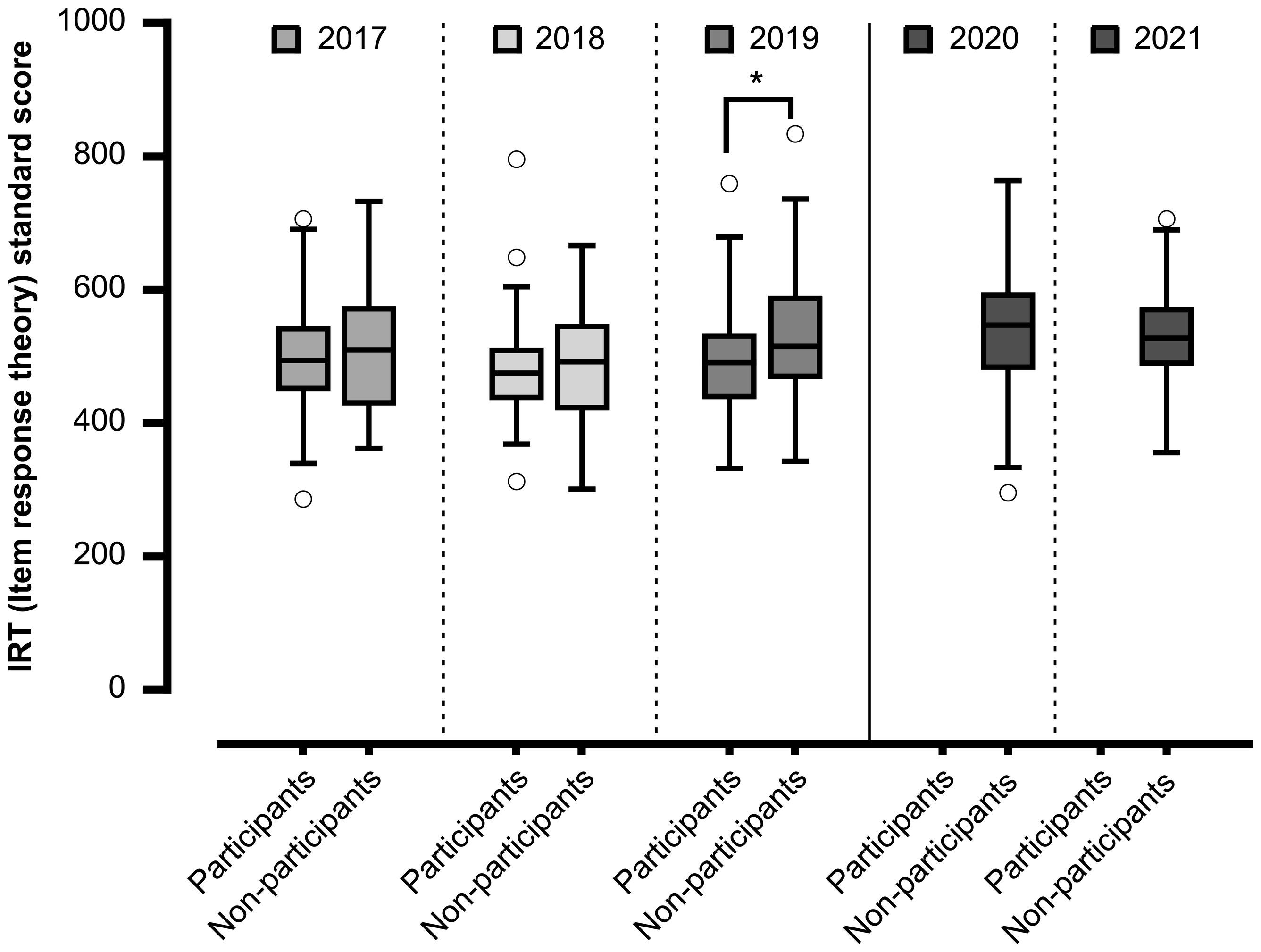

Supplement: S3 Fig — Significance level for *< .05, **< .01. Error bars represent standard deviation. (TIF) [file pone.0265356.s003.tif]
