# Supplementary material for: Effects of COVID-19 on Japanese medical students’ knowledge and attitudes toward e-learning in relation to performance on achievement tests
Source: PLoS One. 2022 Mar 14;17(3):e0265356. doi: 10.1371/journal.pone.0265356 (PMC8920276; doi:10.1371/journal.pone.0265356)
Supplement: S1 Table — (DOCX) [file pone.0265356.s004.docx]

**Supplemental Table 1. Test Sections.**

| **CBT Section** | |
| --- | --- |
| Section 1 | Basic Qualities and Abilities Required in a Physician |
| Section 2 | Society and Medicine/Medical Practice |
| Section 3 | General Issues in Medicine |
| Section 4 | Normal Structure and Function, Pathophysiology, Diagnosis, and Treatment of Each Organ System in the Human Body |
| Section 5 | Systemic Physiological Change, Pathophysiology, Diagnosis, and Treatment |
| Section 6 | Basis of Medical Practice |
