# Supplementary material for: Effects of COVID-19 on Japanese medical students’ knowledge and attitudes toward e-learning in relation to performance on achievement tests
Source: PLoS One. 2022 Mar 14;17(3):e0265356. doi: 10.1371/journal.pone.0265356 (PMC8920276; doi:10.1371/journal.pone.0265356)
Supplement: S2 Table — (DOCX) [file pone.0265356.s005.docx]

| **Supplemental Table 2. Summary of the Kruskal–Wallis test** **of CBT sections** **test score comparison, 2017-2021.** | | | | | |
| --- | --- | --- | --- | --- | --- |
| ***Computer-based achievement (CBT) test***  ***Test section*** | | *χ²* | *df* | *p* | *ε²* |
|  | Section 1 | 15.53 | 4 | .004 | .023 |
|  | Section 2 | 16.82 | 4 | .002 | .025 |
|  | Section 3 | 14.17 | 4 | .007 | .021 |
|  | Section 4 | 22.71 | 4 | <.001 | .034 |
|  | Section 5 | 16.31 | 4 | .003 | .024 |
|  | Section 6 | 5.24 | 4 | .263 | .008 |
| Note. Mean difference is significant at the 0.05 level. | | | | | |
