# Supplementary material for: Effects of COVID-19 on Japanese medical students’ knowledge and attitudes toward e-learning in relation to performance on achievement tests
Source: PLoS One. 2022 Mar 14;17(3):e0265356. doi: 10.1371/journal.pone.0265356 (PMC8920276; doi:10.1371/journal.pone.0265356)
Supplement: S3 Table — (DOCX) [file pone.0265356.s006.docx]

| **Supplemental Table 3. Dunn‑Bonferroni tests comparisons of CBT sections to 2020 and 2021** | | | | | | | | |
| --- | --- | --- | --- | --- | --- | --- | --- | --- |
| ***Computer-based achievement (CBT) test***  ***Test section*** | |  |  |  | *p* | | | |
|  |  |  | N | Mean Rank | vs. 2020 |  | vs. 2021 |  |
|  | CBT section 1 | 2017 | 133 | 304.64 | .853 |  | .005 | ** |
|  |  | 2018 | 126 | 313.98 | 1.000 |  | .023 | * |
|  |  | 2019 | 132 | 322.31 | 1.000 |  | .066 |  |
|  |  | 2020 | 136 | 383.37 | .683 |  |  |  |
|  |  | 2021 | 140 | 342.98 |  |  | .683 |  |
|  | CBT section 2 | 2017 | 133 | 322.52 | .080 |  | 1.000 |  |
|  |  | 2018 | 126 | 317.82 | .049 | * | 1.000 |  |
|  |  | 2019 | 132 | 295.75 | .001 | ** | .296 |  |
|  |  | 2020 | 136 | 346.29 | 1.000 |  |  |  |
|  |  | 2021 | 140 | 383.59 |  |  | 1.000 |  |
|  | CBT section 3 | 2017 | 133 | 327.15 | 1.000 |  | .505 |  |
|  |  | 2018 | 126 | 288.45 | .080 |  | .004 | ** |
|  |  | 2019 | 132 | 326.16 | 1.000 |  | .461 |  |
|  |  | 2020 | 136 | 372.97 | 1.000 |  |  |  |
|  |  | 2021 | 140 | 351.04 |  |  | 1.000 |  |
|  | CBT section 4 | 2017 | 133 | 329.13 | 1.000 |  | .055 |  |
|  |  | 2018 | 126 | 282.27 | .338 |  | <.001 | *** |
|  |  | 2019 | 132 | 327.78 | 1.000 |  | .046 | * |
|  |  | 2020 | 136 | 394.35 | .075 |  |  |  |
|  |  | 2021 | 140 | 332.42 |  |  | .075 |  |
|  | CBT section 5 | 2017 | 133 | 324.47 | .936 |  | .861 |  |
|  |  | 2018 | 126 | 282.08 | .006 | ** | .005 | ** |
|  |  | 2019 | 132 | 330.05 | 1.000 |  | 1.000 |  |
|  |  | 2020 | 136 | 364.80 | 1.000 |  |  |  |
|  |  | 2021 | 140 | 363.59 |  |  | 1.000 |  |
| Note. Significance values have been adjusted by the Bonferroni correction for multiple tests. Significance level for * <.05, ** <.01, and *** <.001. | | | | | | | | |
